# Dicarboxylic esters: Useful tools for the biocatalyzed synthesis of hybrid compounds and polymers

**DOI:** 10.3762/bjoc.11.174

**Published:** 2015-09-09

**Authors:** Ivan Bassanini, Karl Hult, Sergio Riva

**Affiliations:** 1 Istituto di Chimica del Riconoscimento Molecolare, CNR, via Mario Bianco 9, Milano, Italy; 2 School of Biotechnology, Department of Industrial Biotechnology, Albanova KTH, Royal Institute of Technology, Stockholm, Sweden

**Keywords:** biocatalysis, dicarboxylic acids, lipase, polyesters, regioselectivity

## Abstract

Dicarboxylic acids and their derivatives (esters and anhydrides) have been used as acylating agents in lipase-catalyzed reactions in organic solvents. The synthetic outcomes have been dimeric or hybrid derivatives of bioactive natural compounds as well as functionalized polyesters.

## Introduction

The finding that enzymes can work in organic solvents has significantly expanded the scope of preparative scale biocatalyzed transformations [1–4]. An uncountable number of reports have been published on this topic since the eighties of the last century, the vast majority of them dealing with the synthetic exploitation of hydrolases [5–6].

It was found that reactions that are thermodynamically unfavorable in water, like esterifications, transesterifications (transacylations) and amidations, can be efficiently catalyzed by lipases and proteases in organic solvents. Moreover, both substrates and acylating agents’ scope could be significantly expanded. Lipases, whose natural substrates are fatty acid triglycerides, and proteases, enzymes acting on peptides and proteins, were found to be able to catalyze, i.e., the esterifications of sugars and steroids, using acylating agents different from simple aliphatic acids [7–9]. Specifically, years ago Dordick and coworkers proposed the so-called ‘combinatorial biocatalysis’ as an approach to easily produce small libraries of derivatives of bioactive natural compounds using a panel of different acylating agents and hydrolases [10–12].

Among the great number of investigated acyl donors, activated esters of dicarboxylic acids have been found to be particularly versatile for the production of bifunctionalized compounds. As it will be discussed in the following paragraphs, these molecules have allowed the synthesis of dimeric or hybrid derivatives of bioactive natural compounds as well as the biocatalyzed production of functionalized polyesters.

## Review

### 1. Synthetic exploitation of dicarboxylic esters

#### a) Synthesis of activated esters

In most of the biocatalyzed transesterification reactions, ‘activated’ esters are usually employed in order to make the reactions irreversible thanks to the release of alcohols that are poor nucleophiles (halogenated derivatives of ethanol, vinyl or isopropenyl alcohol) [13–15]. This has been also the case with several reports on the use of dicarboxylic acid derivatives.

**Scheme 1:**
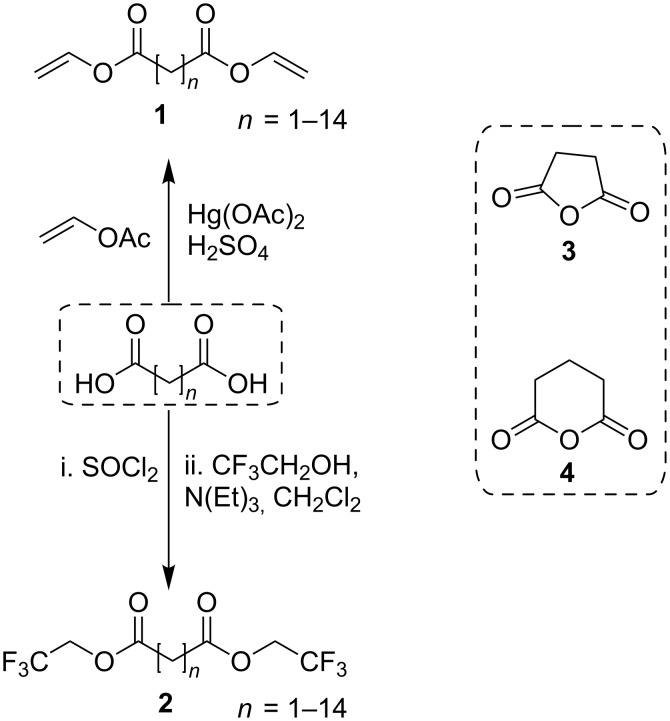
Activated derivatives of dicarboxylic acids.

Accordingly, vinyl diesters (**1**) and trifluoroethyl diesters (**2**) have been synthesized following standard procedures [16]. Moreover, succinic (**3**) and glutaric anhydride (**4**) could be used as acylating agents in controlled biocatalyzed reactions (Scheme 1) [17–18].

#### b) Regioselective enzymatic acylation of natural products.

Natural products are traditionally classified into groups of substances (terpenes, alkaloids, amino acids, lipids, etc), depending on their biosynthetic origin and on their chemical and structural features [19–21]. The complex structures of most of these molecules along with the presence of multiple functional groups make their chemical manipulation difficult. This inherent “fragility” makes biocatalysis an attractive method for their derivatization. Specifically, glycosides and polyhydroxylated compounds can be selectively acylated at specific hydroxy groups by the action of an activated ester in the presence of a suitable hydrolase in organic solvents [22–23].

**Figure 1:**
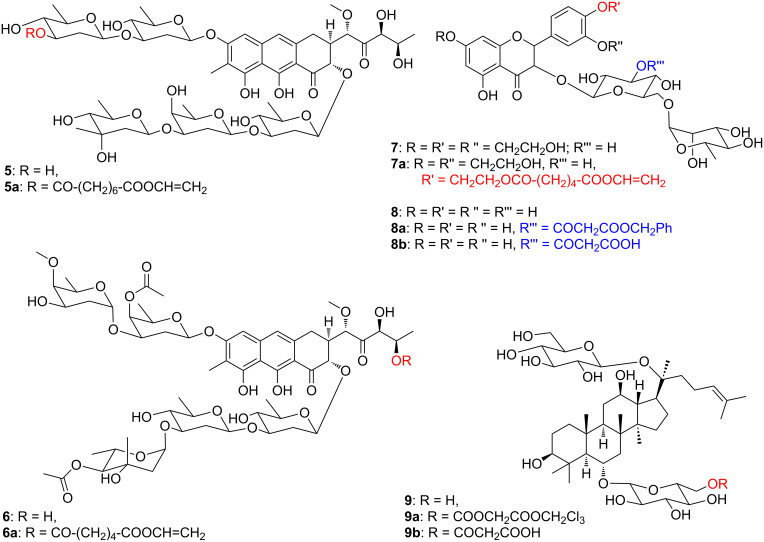
Example of natural compounds selectively acylated with dicarboxylic esters.

Different authors have shown that activated dicarboxylates are also accepted as acyl donors by these enzymes. As an example, Figure 1 shows the products obtained using divinyl adipate in the esterification of the antineoplastic antibiotics mithramycin (**5**) catalyzed by *Candida antarctica* lipase A (CAL-A) and chromomycin A_3_ (**6**) catalyzed by *Candida antarctica* lipase B (CAL-B) [24]. In another report a series of mono-substituted troxerutin esters (**7a**) were synthesized by action of the alkaline protease from *Bacillus subtilis* on **7** [25]. The carboxyacetyl (malonyl) derivative of some flavonoid glycosides (i.e., **8b**) and of ginsenoside Rg1 (**9b**) could be obtained with two-step sequences. The preliminary CAL-B catalyzed acylations of **8** with dibenzyl malonate and of **9** with bis(2,2,2-trichloroethyl)malonate to give the mixed malonyl derivatives **8a** and **9a**, respectively, were followed either by a palladium-catalyzed hydrogenolysis of the benzyl moiety to give **8b** [26], or by a selective chemical removal of 2,2,2-trichloroethanol with Zn/AcOH to give **9b** [27].

#### c) Enzymatic synthesis of symmetric diesters

More recently, symmetric diesters have been synthetized exploiting both the activated extremities of divinyl carboxylates.

**Figure 2:**
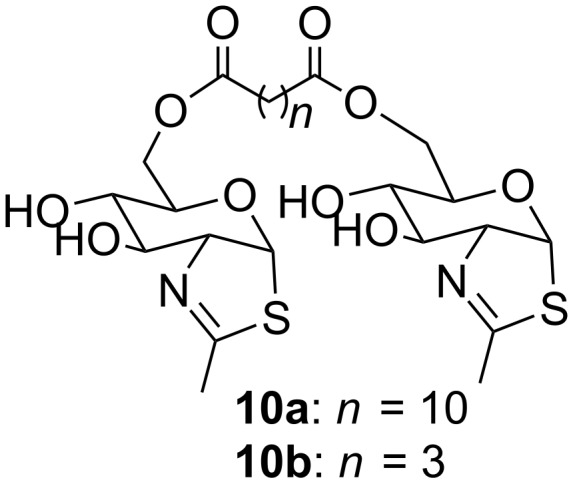
C_6_-dicarboxylic acid diesters derivatives of NAG-thiazoline.

C_6_-dicarboxylic acid diesters derivatives of the thiazoline of *N*-acetylglucosamine (NAG-thiazoline, **10a**,**b**, Figure 2) were prepared and their inhibitor activities towards fungal β-*N*-acetylhexosaminidase evaluated [28].

**Figure 3:**
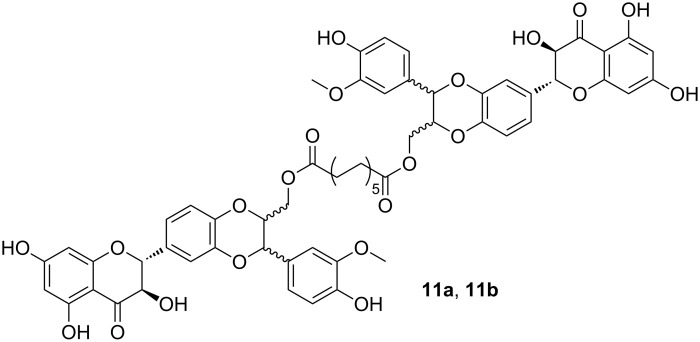
Sylibin dimers obtained by CAL-B catalyzed trans-acylation reactions.

Similarly, dimers of sylibin (**11a**,**b**, Figure 3) and dehydrosylibin, obtained by Novozyme 435-catalyzed acylation with the divinyl esters of dodecanedioc acid, were evaluated in terms of antioxidant activity and cytotoxicity [29].

The obvious hypothesis related to the synthesis of these compounds was that a dimer should be more bioactive than a monomer, but this was not always the case [28–29].

#### d) Enzymatic synthesis of hybrid dimers

**Scheme 2:**
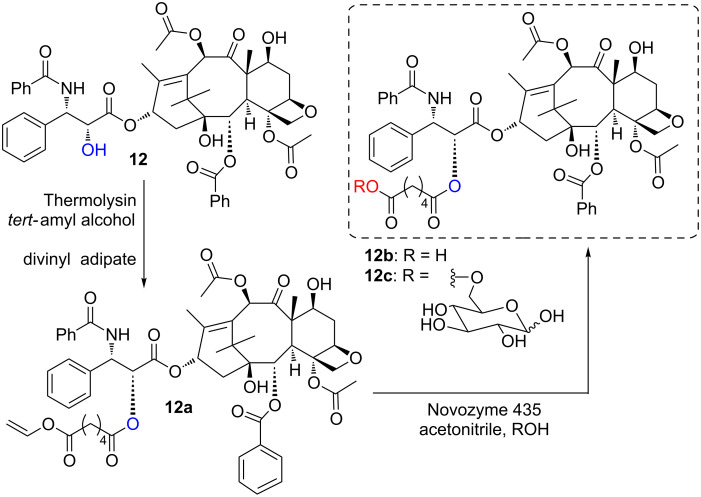
Biocatalyzed synthesis of paclitaxel derivatives.

According to a pioneering paper Dordick linked glucose to paclitaxel with divinyl adipate in a two-step biocatalyzed acylation [30]. As shown in Scheme 2, the protease thermolysin catalyzed the regioselective acylation of the side chain of paclitaxel (**12**) to give the 2’-vinyl adipate **12a** in 60% isolated yields. Novozyme 435-catalyzed elaboration of this intermediate allowed either to hydrolyze the residual vinyl ester to give the carboxyl derivative **12b** (reaction performed in acetonitrile containing 1% H_2_O v/v) or to link it to a sugar, like glucose to give the hybrid compound **12c** (reaction performed in dry acetonitrile containing glucose). Both derivatives were significantly more soluble in aqueous solutions than the parent compound **12**.

**Figure 4:**
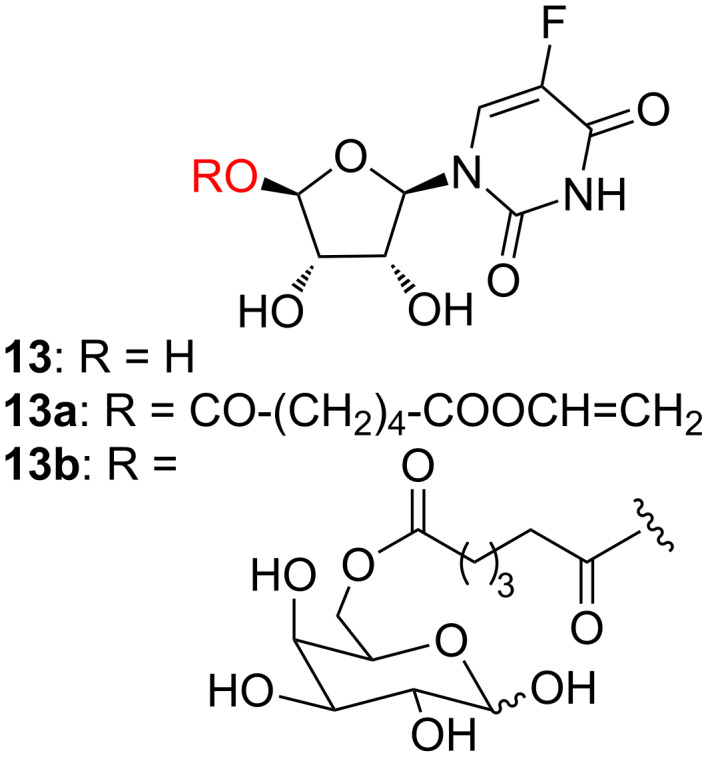
5-Fluorouridine derivatives obtained by CAL-B catalysis.

A similar approach was followed later on by Lin and coworkers, who described the enzymatic esterification of the nucleoside 5-fluorouridine (**13**) and of other polyhydroxylated bioactive molecules with divinyl esters of dicarboxylic acids [31–35]. The monovinyl esters obtained (i.e., **13a**) were then used either to acylate monosaccharides (i.e., galactose to give **13b**) in order to increase the solubility of the parent compounds in aqueous solutions (Figure 4) or as co-monomers in radical (AIBN)-catalyzed polymerizations (see next paragraph).

In recent years linking different bioactive molecules with suitable dicarboxylic acids to prepare hybrid compounds has been receiving more and more attention. The interest is due to the fact that these new substances might show additive activities [36], having improved properties or efficacies compared to the combined use of the respective two parent compounds. This is the so-called ‘dual drug’ strategy [37–41]. For instance [40–41], an increased capacity of inhibiting endothelial cell differentiation and migration (key steps of the angiogenic process) was observed as well as a marked ability to inhibit the polymerization of tubulin in vitro. The same methodology might be applied to direct a drug by conjugation to a molecule binding to a specific receptor on cancer cells. Moreover, by using dicarboxylated linkers with a disulfide bridge, it was possible to generate dynamic libraries of dimeric hybrids based on disulfide exchange reactions in vivo [42–43].

**Scheme 3:**
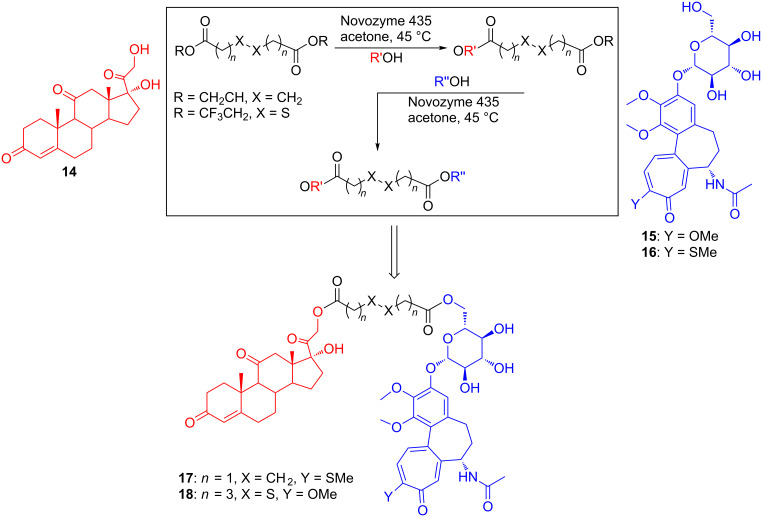
Biocatalyzed synthesis of hybrid diesters **17** and **18**.

All of these compounds were synthesized by (sometimes troublesome) chemical protocols requiring accurate control of the reaction conditions and several protection/deprotection steps. This is avoided using a biocatalyzed approach, as it has been shown exploiting once again the well-known efficiency, selectivity and versatility of CAL-B (Novozyme 435) [16]. As in the previous examples, the mixed esters from the first esterification step can be used as acylating agents in the second esterification step. Scheme 3 shows the synthesis of the hybrid compounds **17** and **18**, obtained by linking together a steroid (cortisone, **14**) and an alkaloid (colchicoside, **15**; thiocolchicoside, **16**). Worth of notice the use, among others, of activated esters of dithio-dicarboxylic acids, in **18**.

**Scheme 4:**
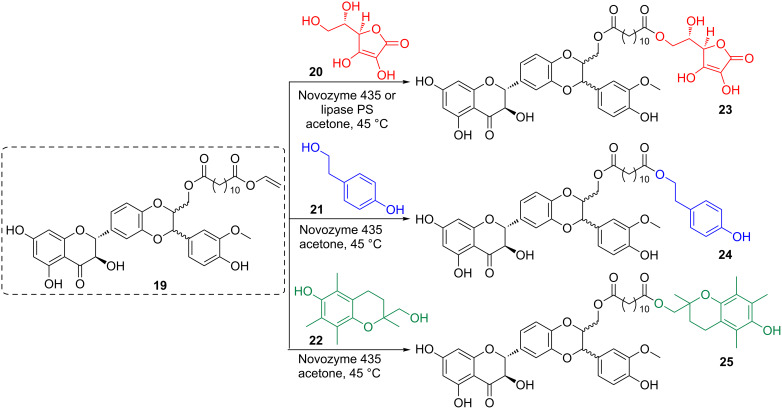
Hybrid derivatives of sylibin.

More recently Kren and coworkers have synthesized hybrid dimeric antioxidants **23**–**25** based on the conjugation of an acylated sylibin derivative (**19**) with *L*-ascorbic acid (**20**), tyrosol (**21**) and trolox alcohol (**22**) (Scheme 4) [44]. These compounds proved to have excellent electron donor, antiradical, antioxidant as well as cytoprotective abilities.

**Figure 5:**
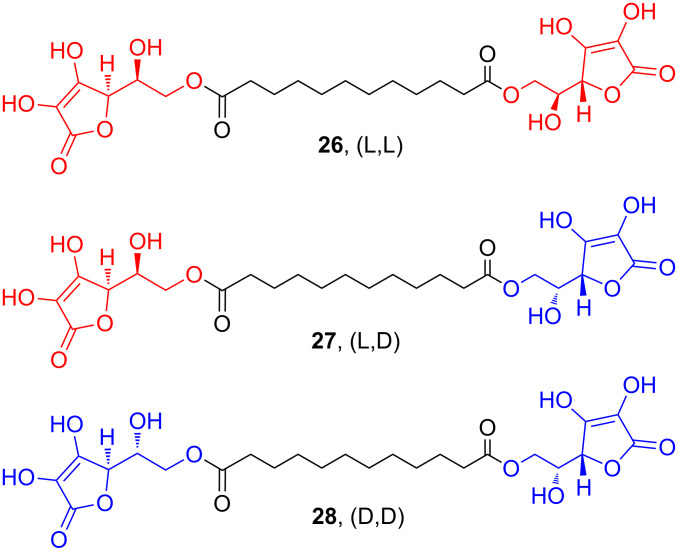
Bolaamphiphilic molecules containing (L)- and/or (D)-isoascorbic acid moieties.

Moreover, in a different research area, studying the supramolecular behavior of bolaamphiphile molecules, it has been reported that polyhydroxylated compounds linked via a dicarboxylic chain (like the symmetric vitamin C-based bolaamphiphile **26**, L,L) give origin to regular structures [45]. The previously described biocatalyzed approach allowed the synthesis of an asymmetric dimer combining L-ascorbic acid and D-isoascorbic acid (**27**, L,D), which behaved significantly differently in terms of supramolecular structure when compared to the symmetric dimers **26** (L,L) and **28** (D,D) (Figure 5) [46].

More recently, Gross and coworkers have described the synthesis of “sweet silicones” by Novozyme 435-catalyzed formation of ester bonds between organosilicon carboxylic diacids and the primary OH’s of 1-*O*-alkyl glucopyranosides [47].

### 2. Enzymatic synthesis of polyesters

The interest in the biocatalyzed synthesis of polyester started at the very beginning of the use of lipases in organic solvents. In 1984 Okumura et al. [48] produced oligomers of several dicarboxylic acids (C_6_ to C_14_) in combination with several diols (C_2_ and C_3_). Since then the use of lipase-catalyzed preparation of polymers has grow very much and has been reviewed many times (see for example Zang et al. [49], Kobayashi and Makino [50], Gross et al. [51]). Nowadays lipases are not only used to achieve simple polycondensation reactions, but are exploited due to their chemo-, stereo- and enantioselectivity. In addition, they are seen as environmentally friendly alternative to traditional polymerization methods [52].

Binns et al. summarized the attempts to scale up synthesis of polyesters by enzyme catalyzed polycondensation of adipic acid and hexane-1,6-diol in a very well-worth reading article [53]. They discussed the very slow progress in achieving high molecular weight polymers and concluded that removal of the leaving group, water, to draw the equilibrium towards polymerization, and the reversal nature of lipase catalysis are two main obstacles. Others have pointed out the latter also [54]. Often a two-step procedure has been used, an initial polymerization to achieve oligomers followed by a second step at higher temperature and/or lower pressure. The synthesis of oligomers and short telechelics (oligomers with functionalized ends) avoids much of the problems and afford better reaction rates.

**Figure 6:**
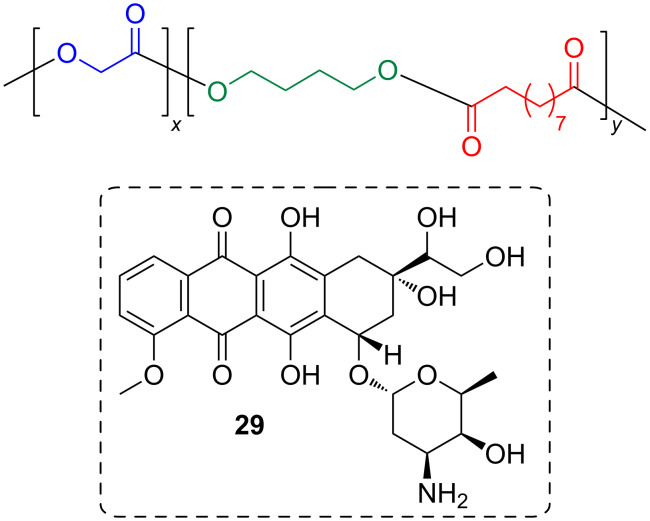
Doxorubicin (29) trapped in a polyester made of glycolate, sebacate and 1,4-butandiol units.

Yang et al. polymerized ethyl glycolate with diethyl sebacate and 1,4-butandiol. For this, they used CAL-B in a two step synthesis, started at a low vacuum and then increased the vacuum to drive the reaction to completion [55]. The dicarboxylic acid and the diol were employed in equal molar amounts, while the amount of ethyl glycolate was varied. Polymers with a high molecular weight (12–18000 Dalton) were obtained (Figure 6). Nano particles of the polymer were used for a controlled slow release of the drug doxorubicin (**29**) trapped in this material.

**Figure 7:**
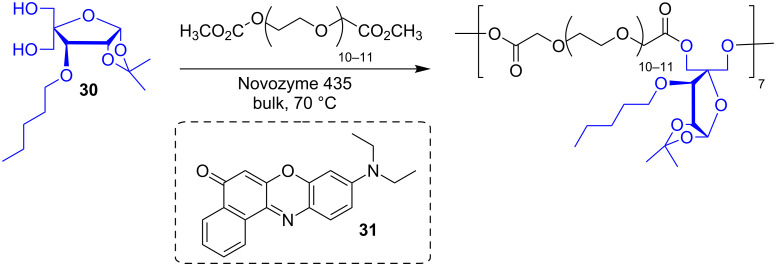
Polyesters containing functionalized pentofuranose derivatives.

Bhatia et al. used Novozyme 435 to make polymers from functionalized pentofuranose derivatives (i.e, **30**) and PEG-600 dicarboxylic acid dimethyl ester [56]. The obtained polymers formed supramolecular aggregates with diameters between 120 and 250 nm, which were able to encapsulate Nile red (**31**) that was used as a model of a drug compound (Figure 7).

**Figure 8:**
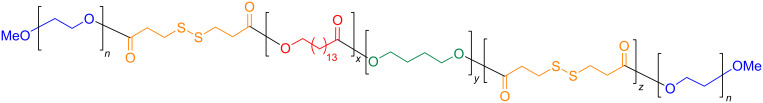
Polyesters containing disulfide moieties.

Copolymers containing disulfide groups in the main chain were synthesized from 3,3´-dithiodipropionic acid dimethyl ester in combination with pentadecalactone and 1,4-butandiol (Figure 8) [57]. When MeO-PEG-OH was used as chain terminator amphiphilic copolymers were formed. The hydrophobicity of the polymer could easily be changed by the content of the lactone. The copolymers had low toxicity and formed aggregates that could be used as nano-containers of drugs. Reduction of the disulfides caused swelling of the aggregates and fast release of incorporated drugs.

**Figure 9:**
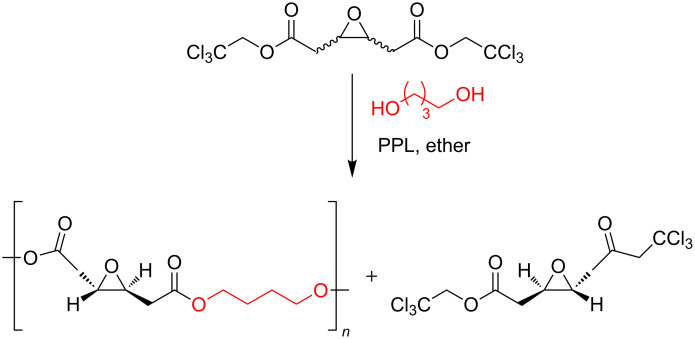
Polyesters containing epoxy moieties.

An early attempt to use dicarboxylic acids with an additional functional group was done by Wallace and Morrow [58]. They used the activated 2,2,2-trichloroethyl diester of (±)-3,4-epoxyadipic acid. The stereoselectivity of porcine pancreatic lipase discriminated between the two enantiomers and afforded the chiral (−)-polyester with molecular weight of 7900 Dalton (Figure 9).

**Figure 10:**
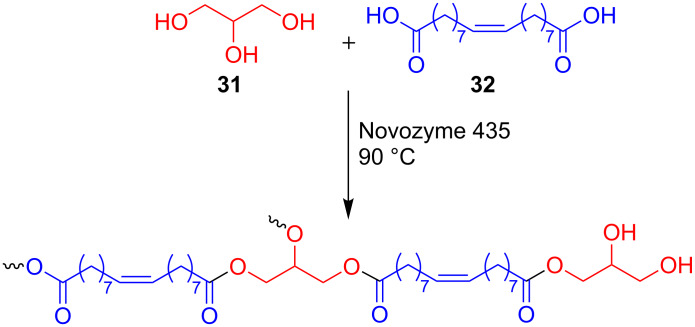
Biocatalyzed synthesis of polyesters containing glycerol.

Yang et al. compared the polymerization of glycerol and a diacid derivative of oleic acid catalyzed by dibutyltin oxide and Novozyme 435 (Figure 10) [59]. Dibutyltin oxide catalysis resulted in cross-linking and gel formation. This was not observed by enzyme catalysis, presumably due to steric hindrance which may be imposed by the active site of the enzyme.

Symmetrical long-chain (C_18_, C_20_ and C_26_) unsaturated or epoxidized dicarboxylic acids were polycondensated with 1,3-propanediol or 1,4-butanediol using CAL-B [60]. At high temperature (70 °C) a number of polyester combinations could be synthesized. Propandiol afforded polymers with rather moderate molecular weights (2000–3000 Dalton), while with butandiol polyesters with higher molecular weights (8000–12000 Dalton) were obtained. Interestingly, the polymers carried functional groups in the chain that could be used for further modifications.

**Figure 11:**
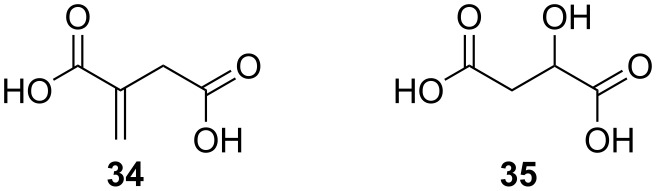
Iataconic (**34**) and malic (**35**) acid.

For polymer synthesis involving environmentally benign chemicals the building blocks succinic acid, itaconic acid (**34**, Figure 11) and butanediol are very attractive. The methylene group in itaconic acid is interesting as a handle for second polymerization or derivatization, but causes steric and reactivity problems in lipase catalysis. Anyhow, Jiang et al. were able to synthesize polyesters with a mix of the two acids used as dimethyl esters. The yield was acceptable if the reaction was run in diphenyl ether and the ratio of itaconate did not exceed 30% [61]. The authors discussed the consequences of the low reactivity of itaconic acid in relation to polymer growth. Another dicarboxylic acid carrying an additional functional group is malic acid (**35**, Figure 11). Yao et al. used (L)-malic acid and adipic acid in different ratios to be polymerized with 1,8-octanediol in a reaction catalyzed by CAL-B [62]. The yield depended on the choice of organic solvent, with isooctane being the best one. Using 10% of enzyme by weight compared to total amount of monomers, molecular sieves to trap the produced water and working at 70 °C, high molecular weight polymers were isolated after 48 h. This was a good example, showing that the selectivity of the lipase-driven polymerization using only the primary alcohols of the diol, and not the secondary hydroxy group of malic acid.

**Figure 12:**
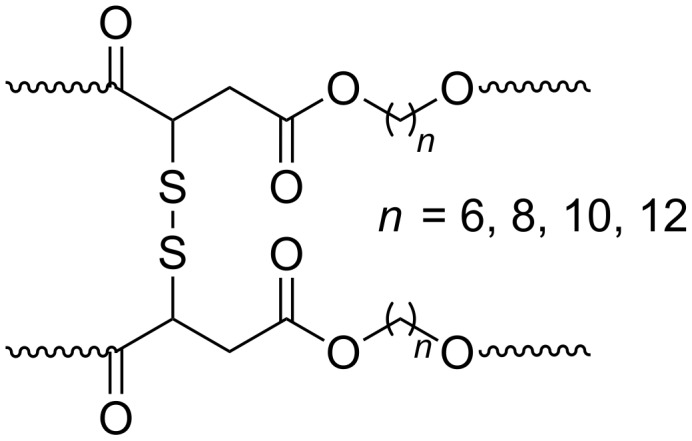
Oxidized poly(hexanediol-2-mercaptosuccinate) polymer.

A few years earlier Kato et al. showed that both enantiomers of dimethyl 2-mercaptosuccinate and 1,6-hexanediol were polymerized by CAL-B, while other lipases failed to give long polymers [63]. In the same article the authors showed that only the (L)-enantiomer of dimethyl malate afforded polymers. A racemate of malate esters gave only short polymers; showing nicely that efficient polymerization of diacids can only be achieved with carboxylic groups of similar reactivity. The poly(hexanediol-2-mercaptosuccinate) could be oxidized by air in DMSO to form a cross-linked insoluble material (Figure 12). In a subsequent paper, the same laboratory prepared different mercaptosuccinate polymers with several diols. In addition they showed that the material cross-linked by air oxidation could be reversibly reduced by tributylphosphine to recover the reduced soluble polymer [64].

**Figure 13:**
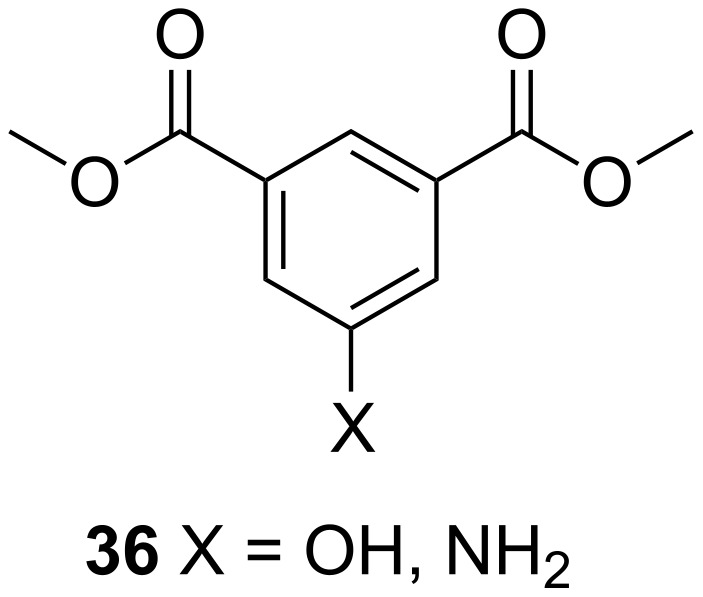
C-5-substituted isophthalates.

In a recent review, Khan et al. summarized the synthesis of polymers based on C-5-substituted isophthalates (**36**, Figure 13) and diols [65]. Using hydroxy or amine groups at C-5 afforded polymers, which could be further modified by chemical means. The synthesized products can find a wide range of applications such as drug/gene delivery systems, flame retardant materials, conducting polymers, controlled release systems, diagnostic agents, and polymeric electrolytes for nano-crystalline solar cells.

**Figure 14:**
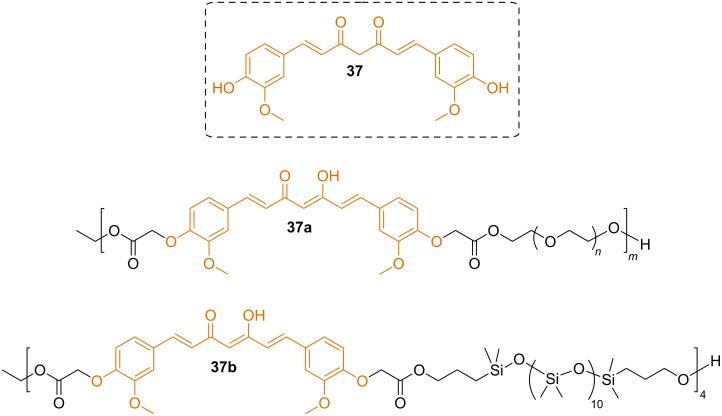
Curcumin-based polyesters.

Curcumin (**37**) was converted to a diester using ethyl α-bromoacetate. The formed diester was copolymerized with PEG using CAL-B (Figure 14). The final product was an effective activator of nuclear factor (erythroid-derived 2)-like 2 (Nrf2) several times better than the free curcumin [66]. The curcumin diester was used in a second polymer synthesis with carbinol (hydroxy) terminated polydimethylsiloxane catalyzed by CAL-B [67]. The curcumin moiety retained its fluorescence properties without quenching in thin films prepared from the polymer. Films exposed to low concentrations of vapors of the explosives DNT and TNT absorbed the explosives and the fluorescence was quenched. Therefore, it was proposed that the films can be used as sensors for these explosives.

**Figure 15:**
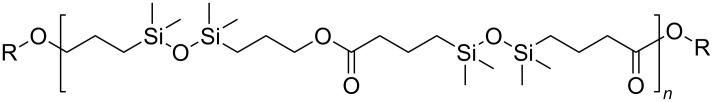
Silylated polyesters.

Frampton et al. synthesised a polyester from the dimethyl ester of 1,3-bis(3-carboxypropyl)-1,1,3,3-tetramethyldisiloxane and 1,3-bis(3-hydroxypropyl)-1,1,3,3-tetramethyldisiloxane (Figure 15) using CAL-B. They obtained the polymers as colorless viscous liquids after evaporation of ether used to extract the polymer from the enzyme beads [68].

#### a) Dicarboxylic esters in combination with functionalized alcohols

**Figure 16:**
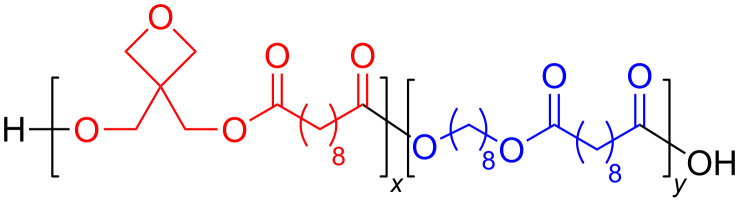
Polyesters containing reactive ether moieties.

The use of diols with additional reactive groups opens up the possibility to synthesize a number of functionalized polymers. For instance, Müller and Frey used 3,3-bis(hydroxymethyl)oxetane in different blends with 1,8-octanediol and sebacic acid to get polymers with a varied content of oxetane groups (Figure 16). Oxetane is a very acid sensitive moiety, but the mild conditions for enzyme catalysis afforded nice polymers. The obtained polymers could be cross-linked by UV light in the presence of the solid photoinitiator Iracure 270 to form hard films [69].

**Figure 17:**
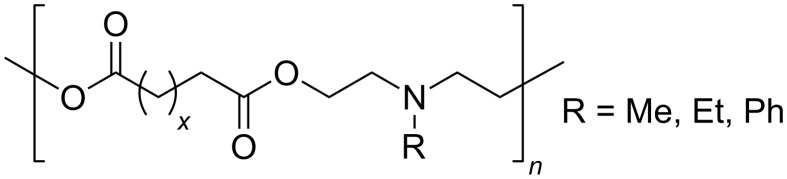
Polyesters obtained by CAL-B-catalyzed condensation of dicarboxylic esters and *N*-substituted diethanolamine.

Several poly(amine-*co*-ester)s were synthesized directly from dicarboxylic acid diesters and *N*-alkyl- or *N*-phenyldiethanolamines. High molecular weights polymers were obtained in a two step procedure catalyzed by CAL-B [70]. Specifically, the obtained polymers from sebacic acid (Figure 17, *x* = 7) and *N*-methyl- or *N*-ethyldiethanolamine proved to form good nanometer-sized complexes with DNA, useful for efficient DNA delivery in gene therapy.

**Figure 18:**
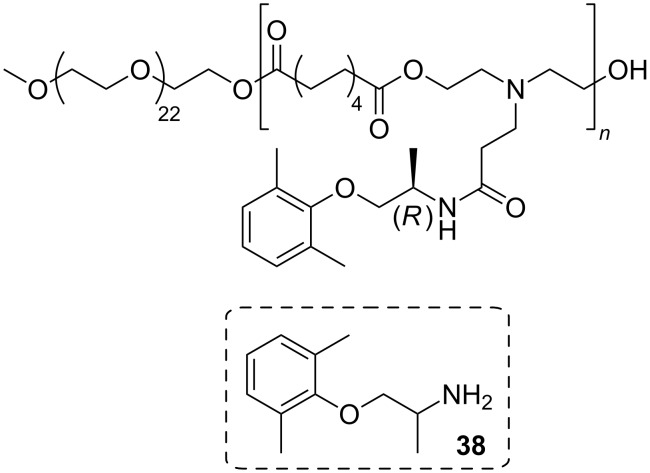
Polyesters comprising mexiletine (**38**) moieties.

Mexiletine (**38**) was incorporated into amphiphilic poly(amine-*co*-ester)s through a two-step lipase catalyzed procedure. Firstly, racemic mexiletine was used in a biocatalyzed kinetic resolution to form the amide with pure (*R*)-amide with methyl 3-(bis(2-hydroxyethyl)amino)propanoate. The formed diol was mixed with an equal molar amount of divinyl sebacate and lipase as a catalyst, after some time methoxypoly(ethylene glycol) was added to react with the remaining vinyl carboxylates to give an amphiphilic polymer. This product self-assembled into nanometer-scale-sized particles in water and could be used for drug delivery (Figure 18) [71].

A few years earlier the same authors used the same principle to synthesize amphiphilic mPEG-block-poly(profenamide-co-ester) copolymers that self-assembled in water and could be used for drug release [72]. As a follow up the same laboratory used triethanolamine and different dimethyl esters of linear dicarboxylic acids to synthesize hyperbranched polymers. With a very high load of CAL-B (20% weight compared to triethanolamine), a long incubation time at 85 °C, and 1–2 mmHg pressure the hyperbranched polymers were isolated [73].

#### b) Amines in combination with dicarboxylic acids

**Figure 19:**
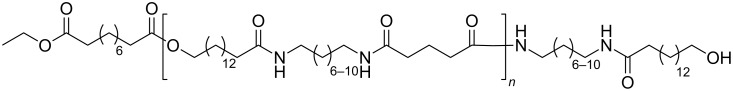
Poly(amide-*co*-ester)s comprising a terminal hydroxy moiety.

Several high molecular weight poly(amide-*co*-ester)s were prepared in a three-step procedure. Significantly high molecular weights were achieved by first reacting pentadecalactone with equal molar amounts of linear diamines. The formed amides, containing one terminal hydroxy and one terminal amino moiety, were further reacted with diethyl sebacate to form high molecular weight poly(amide-*co*-ester)s with a repetitive pattern of amide and ester bonds (Figure 19) [74].

**Figure 20:**
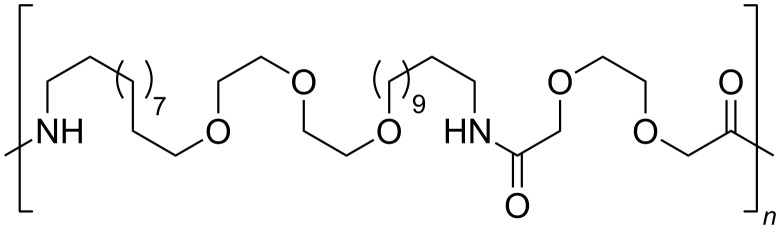
Polymer comprising α-oxydiacid moieties.

The problem of high molecular weights in lipase-catalyzed polyamide synthesis using dicarboxylic acids and diamines has been discussed in several articles. The slow catalytic rate and the insolubility of the formed polymers are two main obstacles. The rate problem was addressed by Poulhès et al. who used an α-oxy diacid derivative (Figure 20), obtaining higher reaction rates, but, unfortunately, lower molecular weights [75]. The observed rate enhancement was presumably an effect of transition state stabilization for the nitrogen inversion in the presence of an oxygen atom in the proximity of the forming amide bond [76].

#### c) Telechelics

Several authors have discussed the difficulty of obtaining high molecular weight polyesters by lipase catalysis. This problem can be circumvented by the synthesis of telechelics, oligomers with functional ends. The synthesis of oligomers avoids the precipitation of polymers during the synthesis. The functional ends of the telechelics can be used in a second step for polymerisation or crosslinking without the lipase. By exploiting the substrate selectivity of lipases it is possible to obtain well-defined telechelics in a one-pot, or even one-step reaction.

**Figure 21:**
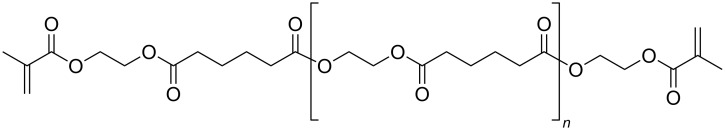
Telechelics with methacrylate ends.

In 1997 Uyama et al. were the first to produce telechelic polyesters from the monomers divinyl sebacate and 12-dodecanolide by lipase PF catalysis. By using 2–3% of the divinyl ester a mixture of telechelic polyesters carrying carboxylic acid ends was achieved [77]. The mixture was probably a result of uncontrolled water content in the incubation. Eriksson et al. used CAL-B to obtain well-defined telechelics in a one-pot polycondensation. The backbone of the telechelics was built from ethylene glycol and divinyl adipate. Specific degrees of polymerisation (4, 8 and 13) were reached by terminating the process with the addition of 2-hydroxyethyl methacrylate. Well-defined telechelics with more than 90% methacrylate ends were used directly in film formation, without any other purification than filtering off the immobilized lipase (Figure 21). The telechelics were either homopolymerized or polymerized in combination with a tetrathiol cross-linker to form strong films under UV irradiation [78].

**Figure 22:**
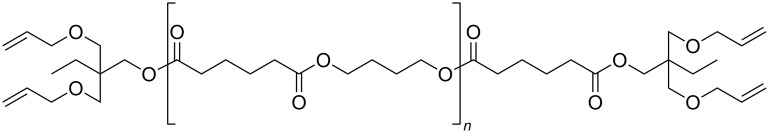
Telechelics with allyl-ether ends.

In a similar approach the same research group synthesized the telechelic tetraallyl ether-poly(butylene adipate) (Figure 22). Each telechelic molecule carried four allyl ether groups, which allowed extensive crosslinking using thiolene chemistry with dithiols or tetrathiols [79].

**Figure 23:**
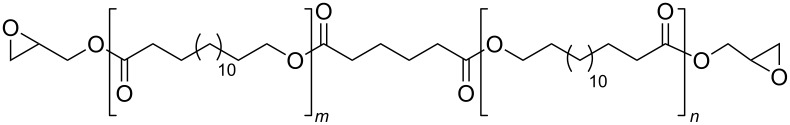
Telechelics with ends functionalized as epoxides.

Through a combination of lipase-catalyzed condensation and ring-opening polymerisation oligomers of pentadecalactone and adipic acid were terminated by glycidol (Figure 23). By changing the stoichiometry of the building blocks, telechelics of different controlled molecular weights could be obtained, which readily polymerized to form films after filtering off the enzyme. The properties of the films depended on the fraction of pentadecalactone and crosslinking density [80].

## Conclusion

In this short review it has been discussed the synthetic potential of dicarboxylic esters in biocatalyzed reactions. Literature examples related to polyesters are significantly more numerous. Nevertheless, as it has been shown in the initial paragraphs, this methodology allows also the facile synthesis of hybrid derivatives of natural compounds with modified physical–chemical properties (i.e., increased water solubility, different supramolecular behavior) and with possible synergic biological activities.
